# Nebulised interferon-β1a (SNG001) in hospitalised COVID-19: SPRINTER phase III study

**DOI:** 10.1183/23120541.00605-2022

**Published:** 2023-03-27

**Authors:** Phillip D. Monk, Jody L. Brookes, Victoria J. Tear, Toby N. Batten, Marcin Mankowski, Tatjana Adzic-Vukicevic, Michael G. Crooks, Davinder P.S. Dosanjh, Monica Kraft, Christopher E. Brightling, Felicity J. Gabbay, Stephen T. Holgate, Ratko Djukanovic, Tom M.A. Wilkinson

**Affiliations:** 1 Synairgen Research Ltd, Southampton, UK; 2 Veramed, Twickenham, UK; 3 tranScrip Ltd, Wokingham, UK; 4 University Clinical Center of Serbia, COVID Hospital Batajnica, Belgrade, Serbia; 5 Respiratory Research Group, Hull York Medical School, University of Hull, Kingston upon Hull, UK; 6 Birmingham and West Midlands Lung Research Unit, University Hospitals Birmingham NHS Foundation Trust, Birmingham, UK; 7 Department of Medicine, College of Medicine, Tucson, AZ, USA; 8 Asthma and Airway Disease Research Center, University of Arizona Health Sciences, Tucson, AZ, USA; 9 Institute for Lung Health, NIHR Leicester Biomedical Research Centre, University of Leicester, Leicester, UK; 10 NIHR Southampton Biomedical Research Centre, Clinical and Experimental Sciences, University of Southampton, Southampton, UK; 11 For a list of the SPRINTER Study Group members, see the supplementary material

## Abstract

**Background:**

Despite the availability of vaccines and therapies, patients are being hospitalised with coronavirus disease 2019 (COVID-19). Interferon (IFN)-β is a naturally occurring protein that stimulates host immune responses against most viruses, including severe acute respiratory syndrome coronavirus 2. SNG001 is a recombinant IFN-β1a formulation delivered to the lungs *via* nebuliser. SPRINTER assessed the efficacy and safety of SNG001 in adults hospitalised due to COVID-19 who required oxygen *via* nasal prongs or mask.

**Methods:**

Patients were randomised double-blind to SNG001 (n=309) or placebo (n=314) once daily for 14 days plus standard of care (SoC). The primary objective was to evaluate recovery after administration of SNG001 *versus* placebo, in terms of times to hospital discharge and recovery to no limitation of activity. Key secondary end-points were progression to severe disease or death, progression to intubation or death and death.

**Results:**

Median time to hospital discharge was 7.0 and 8.0 days with SNG001 and placebo, respectively (hazard ratio (HR) 1.06 (95% CI 0.89–1.27); p=0.51); time to recovery was 25.0 days in both groups (HR 1.02 (95% CI 0.81–1.28); p=0.89). There were no significant SNG001–placebo differences for the key secondary end-points, with a 25.7% relative risk reduction in progression to severe disease or death (10.7% and 14.4%, respectively; OR 0.71 (95% CI 0.44–1.15); p=0.161). Serious adverse events were reported by 12.6% and 18.2% patients with SNG001 and placebo, respectively.

**Conclusions:**

Although the primary objective of the study was not met, SNG001 had a favourable safety profile, and the key secondary end-points analysis suggested that SNG001 may have prevented progression to severe disease.

## Abstract 2

**Although the primary objective was not met, potentially due to improved standard of care, SNG001 had a favourable safety profile and key secondary end-points analysis suggested that SNG001 may have impacted progression of COVID-19 to severe disease** https://bit.ly/3huTDX9

## Introduction

The severe acute respiratory syndrome coronavirus 2 (SARS-CoV-2) pandemic has highlighted the impact of respiratory viruses on mortality and morbidity, and their resulting pressures on healthcare provision. Despite the availability of vaccines and therapies, patients continue to be hospitalised with, and die from, coronavirus disease 2019 (COVID-19) [1, 2], emphasising the need for treatments with novel mechanisms of action, especially for hospitalised patients.

Interferon (IFN)-β is a naturally occurring protein that stimulates immune responses critical for the development of host protection against most viruses, including SARS-CoV-2 [3–6]. It is produced as an immediate local response to viral infection and results in antiviral protein production that limits viral replication [7–9]. SARS-CoV-2 suppresses IFN-β release [10, 11], allowing viral spread throughout the respiratory tract. Furthermore, patients with deficient IFN responses, *e.g.* due to genetics, ageing, comorbidities or autoantibodies against type I IFNs (typically IFN-α and -ω, with a minority of patients having antibodies against IFN-β), are at greater risk of severe viral lung disease [12–15]. Importantly, patients hospitalised due to COVID-19 can have prolonged viral shedding (>17 days), especially those with more severe disease [16]. Overall, therefore, evidence points to the potential for enhancing the host's innate immune response by administering IFN-β into the lungs as an effective treatment against COVID-19 [9].

Injectable and subcutaneous formulations of IFN-β have not demonstrated clinically meaningful effects in COVID-19 [17], possibly because they result in low IFN-β concentrations within the lungs. SNG001 is a unique formulation of recombinant IFN-β1a that contains few excipients and has near-neutral pH, making it suitable for inhaled administration. The aim of delivery *via* nebuliser is to achieve a high local concentration within the lower respiratory tract, the site of SARS-CoV-2 infection [18]. Inhaled SNG001 has been shown to upregulate antiviral biomarker levels in the lungs of patients with COPD or asthma [19–21] and to have potent *in vitro* antiviral activity against SARS-CoV-2, including a number of variants of concern [22].

In addition to the scientific evidence suggesting the need to restore robust IFN responses, a phase II study conducted early in the course of the pandemic in patients hospitalised with COVID-19 showed that those who received SNG001 were more likely to improve, and recovered more rapidly, than those who received placebo [23]. This provided the rationale for the current phase III study (SPRINTER; SARS-CoV-2: Phase III TRial of Inhaled INTERferon-β Therapy), the aim of which was to evaluate the efficacy and safety of SNG001 in patients hospitalised due to COVID-19 who required oxygen therapy *via* nasal prongs or mask, but who did not need high-flow oxygen or ventilatory support.

## Material and methods

### Study design

In this double-blind, placebo-controlled, international study, patients were randomly assigned to SNG001 or matching placebo once daily *via* vibrating mesh nebuliser (Aerogen Solo; Dangan, Galway, Ireland) for 14 days. The effect of study treatment was assessed on top of current local standard of care (SoC), with no limitation on concomitant medications for the treatment of COVID-19 or vaccination. Patients discharged during the 14-day treatment phase completed treatment at home, with study staff providing support *via* telephone or video call. Patients were followed for up to 90 days.

The study was approved by the independent ethics committees or research boards at each institution, performed in accordance with the principles of the Declaration of Helsinki and the International Conference on Harmonization notes for guidance on Good Clinical Practice (ICH/CPMP/135/95), and registered in the ISRCTN registry (ISRCTN85436698). The protocol was amended four times (supplementary table S1).

### Patients

The study recruited males or females, ≥18 years of age, hospitalised due to COVID-19 and requiring oxygen *via* nasal prongs or mask. Key exclusion criteria were ongoing SARS-CoV-2 infection that had lasted ≥3 weeks, previous SARS-CoV-2 infection, noninvasive ventilation, high-flow nasal oxygen therapy, endotracheal intubation and invasive mechanical ventilation (for the full list of criteria, see the supplementary material). Patients provided informed consent prior to any study-related procedure.

### Study procedures

Patients were assigned to treatment according to a randomisation schedule, with investigators and patients blinded to treatment by matching placebo. Study medication was presented in two pre-filled syringes, each containing 0.65 mL solution (for SNG001 this contained 12 MIU·mL^−1^ IFN-β1a). SNG001 dose selection was based on prior clinical and animal data ([23] and data on file). In the previous phase II study [23], SNG001 was delivered using the I-neb nebuliser (Philips Respironics, Tangmere, UK), with the administered dose delivering a lung dose of 3.8 MIU; when delivered *via* the vibrating mesh nebuliser, the same administered dose is predicted to provide a lung dose of ∼5 MIU (data on file). During nebulisation, supplemental oxygen could be administered *via* nasal cannula; if this was insufficient, additional oxygen could be administered through the nebuliser's oxygen port.

Prior to administration of the first dose, and daily up to day 28, the following assessments were completed: World Health Organization (WHO) Ordinal Scale of Clinical Improvement (OSCI) [24], Breathlessness, Cough and Sputum Scale (BCSS) [25], National Early Warning Score 2 (NEWS2; only while hospitalised) [26], and EuroQol 5-Dimension 5-Level (EQ-5D-5L; on days 7, 15 and 28 only). COVID-19 symptoms were assessed from days 1 to 35, with WHO OSCI and EQ-5D-5L also completed daily from days 29 to 35. See the supplementary material for details. Adverse events were recorded up to 28 days after the patient's last dose, with physical examinations and vital signs assessed daily while the patient was in hospital.

### Outcomes

The primary objective was to evaluate recovery after administration of SNG001 compared with placebo. The two primary end-points were time to hospital discharge (WHO OSCI score ≤2, sustained for ≥7 days and without readmission prior to day 35) and time to recovery to no limitation of activity (WHO OSCI score ≤1, sustained for ≥7 days). The study was to be considered successful if SNG001 was statistically superior to placebo for at least one of the primary end-points. The three key secondary end-points were: progression to severe disease or death (WHO OSCI score ≥5); progression to intubation or death (WHO OSCI score ≥6); and death, all assessed up to 35 days after first dose.

Other secondary end-points were: the proportion of patients recovering (WHO OSCI score ≤1, sustained for ≥7 days), discharged from hospital and with an improvement in WHO OSCI, each at days 7, 14, 21 and 28; changes in BCSS total score and individual domains during the treatment period; changes in NEWS2 during the hospitalisation period; daily assessment of COVID-19 symptoms; limitation of usual activities; and quality of life measured using EQ-5D-5L. Safety and tolerability were assessed throughout the study by recording vital signs, adverse events, concomitant medications and immunogenicity.

### Statistical analysis

A sample size of 610 patients (n=305 per treatment arm) was estimated to provide ≥90% power to detect a hazard ratio (HR) of 1.45 in time to hospital discharge and a HR of 1.70 in time to recovery, with ≥95% power to declare statistical significance on at least one of the primary end-points. This sample size was calculated using a global two-sided a level of 0.05, adjusted with the Hochberg procedure to allow for multiple comparisons. The sample size calculation assumed 70% hospital discharge in the placebo arm at day 28, 30% recovery in the placebo arm at day 28 and a dropout rate of 25% over the 28-day evaluation period, with time to dropout exponentially distributed.

The hazard ratios for the two primary end-points were estimated from Cox proportional hazards models with covariates for age, sex, prior duration of COVID-19 symptoms, geographic region and COVID-19 vaccination status, with multiplicity controlled by the Hochberg procedure. For the key secondary end-points, odds ratios were estimated using logistic regression models with the same covariates as the primary analyses. See the supplementary material for the other secondary end-points.

The intention-to-treat (ITT) population, used for the efficacy analyses, comprised all randomised patients. The per-protocol population, used for supportive analyses of the primary and key secondary end-points, comprised all patients in the ITT population who did not have any protocol deviations with an impact on efficacy (see Results and supplementary table S2). The safety population consisted of the ITT population that received at least one dose of study medication.

## Results

**FIGURE 1:**
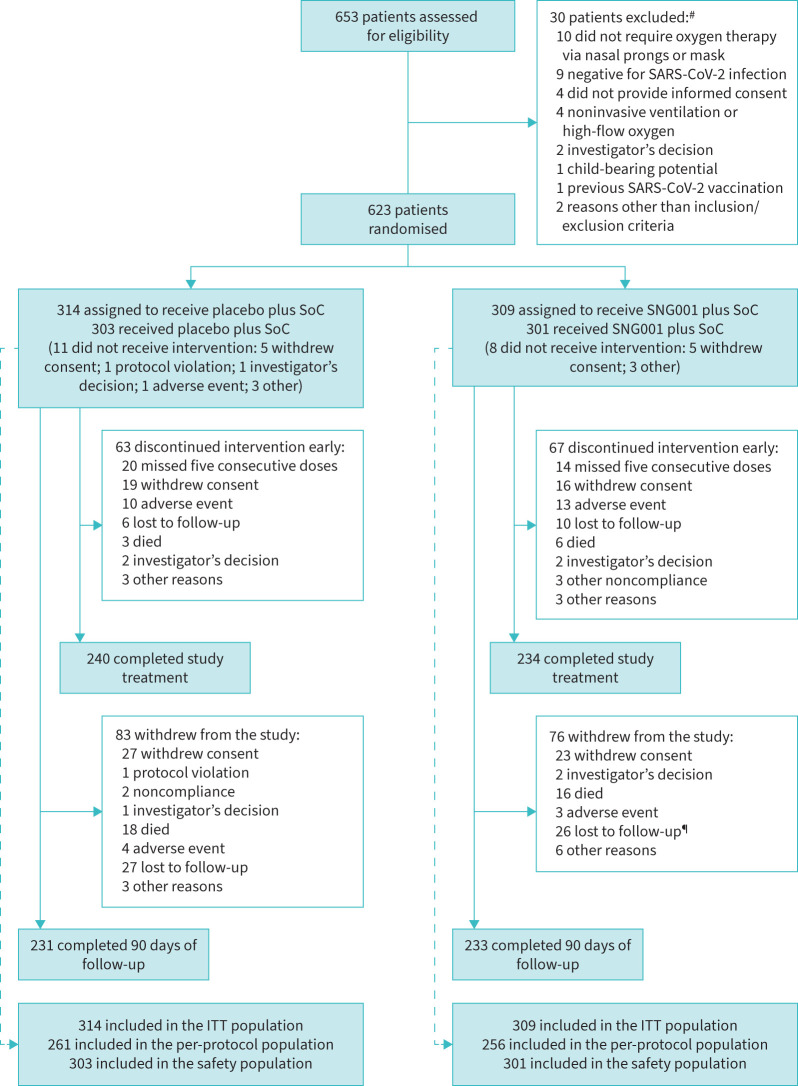
Patient flow through the study. ^#^: patients may be included in more than one category; ^¶^: two patients in the SNG001 plus standard of care (SoC) group were lost to follow-up during the first 35 days but contact was re-established at day 90. ITT: intention-to-treat.

The study was conducted between 12 January 2021 and 10 February 2022 at 111 sites in 17 countries. Of 653 patients screened, 623 were randomised, 309 to receive SNG001 plus SoC and 314 to placebo plus SoC, with 234 and 240 patients, respectively, completing treatment (figure 1). The main reason for exclusion from the per-protocol population was failure to receive at least two doses of study medication in the first 3 days of treatment (supplementary table S2). Baseline demographics and disease characteristics were similar in the two groups (table 1).

**TABLE 1:** Patient baseline demographics and disease characteristics (intention-to-treat population)

	**Placebo plus SoC (n=314)**	**SNG001 plus SoC (n=309)**
**Age, years**	53.7±14.42	52.0±15.19
<40 years	58 (18.5)	66 (21.4)
40–64 years	181 (57.6)	178 (57.6)
≥65 years	75 (23.9)	65 (21.0)
**Male**	208 (66.2)	203 (65.7)
**Race**		
White	215 (68.5)	224 (72.5)
Asian	48 (15.3)	42 (13.6)
Black	6 (1.9)	7 (2.3)
Other/unknown	45 (14.3)	36 (11.7)
**Body mass index, kg·m^−2^**	30.5±7.48	29.6±6.13
≥30 kg·m^−2^	131 (41.7)	112 (36.2)
**Smoking status**		
Current smoker or e-cigarette user	19 (6.1)	15 (4.9)
Ex-smoker	79 (25.2)	76 (24.6)
**Any comorbidity**	158 (50.3)	156 (50.5)
Cancer	16 (5.1)	16 (5.2)
Cerebrovascular disease	8 (2.5)	5 (1.6)
Chronic kidney disease	13 (4.1)	8 (2.6)
Chronic lung disease	20 (6.4)	22 (7.1)
Chronic liver disease	1 (0.3)	1 (0.3)
Diabetes (type I or type II)	52 (16.6)	59 (19.1)
Heart condition	117 (37.3)	117 (37.9)
Mental health disorder	30 (9.6)	27 (8.7)
**Duration of symptoms at randomisation, days**	9.5±3.66	9.6±3.64
**NEWS2 score**	4.3±1.92; n=305	4.3±1.93; n=302
**BCSS total score**	4.6±2.38	4.8±2.54; n=306
**COVID-19 vaccination status**		
Not vaccinated	224 (71.3)	231 (74.8)
Partially vaccinated	30 (9.6)	24 (7.8)
Fully vaccinated	60 (19.1)	54 (17.5)
**COVID-19-related therapy at baseline**		
Remdesivir	64 (20.4)	54 (17.5)
Corticosteroids	275 (87.6)	267 (86.4)
Dexamethasone	229 (72.9)	216 (69.9)

Data are presented as mean±sd or n (%). SoC: standard of care; NEWS2: National Early Warning System-2; BCSS: Breathlessness, Cough and Sputum Scale.

### Outcomes

The median time to hospital discharge in the ITT population was 7.0 (95% CI 7.0–8.0) days with SNG001 plus SoC compared with 8.0 (95% CI 7.0–9.0) days with placebo plus SoC, with a nonsignificant HR of 1.06 (95% CI 0.89–1.27); p=0.51. Results were similar in the per-protocol population (HR 1.02 (95% CI 0.84–1.23); p=0.85). The median time to recovery to no limitation of activity in the ITT population was 25.0 (22.0, upper CI not calculable) days in both treatment groups, with a nonsignificant HR of 1.02 (95% CI 0.81–1.28); p=0.89. Again, per-protocol population results were similar (HR 1.01 (95% CI 0.79–1.29); p=0.93).

SNG001 plus SoC *versus* placebo plus SoC differences for the key secondary end-points were not statistically significant. The proportion of patients who progressed to severe disease or death by day 35 was 25.7% lower (OR 0.71 (95% CI 0.44–1.15); p=0.161) in the SNG001 plus SoC group compared with the placebo plus SoC group in the ITT population and 36.0% lower (OR 0.63 (95% CI 0.35–1.13); p=0.119) in the per-protocol population (table 2). Similarly, the proportions of patients who were intubated or died, or who died within 35 days, were lower in the SNG001 plus SoC group compared with the placebo plus SoC group. For the other secondary end-points, there were no prominent differences between the two treatment groups (supplementary tables S3 and S4, and supplementary figures S1–S5).

**TABLE 2:** Key secondary end-points, assessed up to day 35

**End-point**	**Intention-to-treat**		**Per-protocol**	
	**Placebo plus SoC (n=314)**	**SNG001 plus SoC (n=309)**	**Placebo plus SoC (n=261)**	**SNG001 plus SoC (n=256)**
**Patients who progressed to severe disease or death within 35 days**				
n (%)	45 (14.4)	33 (10.7)	32 (12.3)	20 (7.8)
OR (95% CI); p-value	0.71 (0.44–1.15); 0.161		0.63 (0.35–1.13); 0.119	
Relative risk reduction (%)	25.7		36.0	
**Patients who progressed to intubation or death within 35 days**				
n (%)	23 (7.3)	20 (6.5)	15 (5.7)	10 (3.9)
OR (95% CI); p-value	0.85 (0.45–1.61); 0.610		0.76 (0.34–1.72); 0.512	
Relative risk reduction (%)	11.6		32.0	
**Patients who died within 35 days**				
n (%)	17 (5.4)	14 (4.5)	12 (4.6)	7 (2.7)
OR (95% CI); p-value	0.79 (0.38–1.67); 0.544		0.65 (0.26–1.64); 0.363	
Relative risk reduction (%)	16.3		40.5	

SoC: standard of care. Odds ratios and relative risk reductions are SNG001 plus SoC *versus* placebo plus SoC.

**FIGURE 2:**
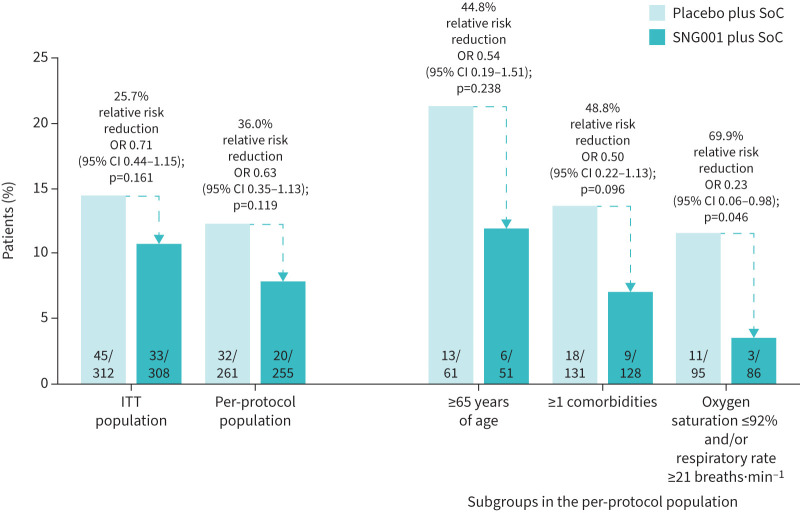
Results of *post hoc* subgroup analyses: patients who progressed to severe disease or death within 35 days (World Health Organization Ordinal Scale of Clinical Improvement score ≥5). SoC: standard of care; ITT: intention-to-treat.

In order to gain further insight into subgroups that may be responsive to treatment, a *post hoc* analysis of progression to severe disease or death within 35 days (WHO OSCI score ≥5) was conducted, with patients subgrouped by baseline parameters that are associated with an increased risk of severe COVID-19: increased age (≥65 years), ≥1 comorbidities and poor respiratory function (oxygen saturation ≤92% and/or respiratory rate ≥21 breaths·min^−1^ while on supplemental oxygen). This analysis was conducted in the per-protocol population so as to focus on the patients who had received study medication and clinical care according to the protocol stipulations. The odds ratios were higher in all subgroups compared with the overall per-protocol population, especially in patients with poor respiratory function in whom a significant (69.9%; p=0.046) reduction was observed (figure 2). Given the *post hoc* nature of these results, with multiplicity not protected, these data should be considered exploratory.

### Safety

Overall, a similar proportion of patients in the two treatment groups experienced adverse events and the most common events were similar in the two groups (table 3). The majority were not considered related to treatment and were mild or moderate in severity. Fewer patients in the SNG001 plus SoC group experienced severe or serious adverse events than in the placebo plus SoC group. Of note, pulmonary embolism adverse events were only seen in the placebo group, with five considered serious. There were no marked differences between the two groups in any of the other safety parameters.

**TABLE 3:** Treatment-emergent adverse events (AEs), overall and most common MedDRA preferred terms^#^

	**Placebo plus SoC (n=303)**	**SNG001 plus SoC (n=301)**
**Any AE**	251 (82.8)	251 (83.4)
Headache	61 (20.1)	71 (23.6)
Productive cough	72 (23.8)	70 (23.3)
Myalgia	60 (19.8)	60 (19.9)
Rhinorrhoea	54 (17.8)	58 (19.3)
Oropharyngeal pain	47 (15.5)	55 (18.3)
Arthralgia	54 (17.8)	54 (17.9)
Wheezing	35 (11.6)	45 (15.0)
Fatigue	40 (13.2)	39 (13.0)
Cough	28 (9.2)	39 (13.0)
Chest pain	52 (17.2)	37 (12.3)
Dyspnoea	42 (13.9)	30 (10.0)
**Any AE related to treatment**	77 (25.4)	68 (22.6)
Headache	16 (5.3)	17 (5.6)
**Any AE leading to discontinuation of study treatment**	23 (7.6)	24 (8.0)
COVID-19 pneumonia	1 (0.3)	5 (1.7)
COVID-19	3 (1.0)	2 (0.7)
Respiratory failure	3 (1.0)	5 (1.7)
Acute respiratory failure	3 (1.0)	0
**Any severe AE**	42 (13.9)	34 (11.3)
Respiratory failure	9 (3.0)	7 (2.3)
COVID-19	5 (1.7)	7 (2.3)
COVID-19 pneumonia	5 (1.7)	7 (2.3)
Pneumonia	0	3 (1.0)
Dyspnoea	0	3 (1.0)
Acute respiratory failure	7 (2.3)	1 (0.3)
**Any serious AE**	55 (18.2)	38 (12.6)
COVID-19	8 (2.6)	11 (3.7)
Respiratory failure	9 (3.0)	9 (3.0)
COVID-19 pneumonia	8 (2.6)	8 (2.7)
Acute kidney injury	2 (0.7)	3 (1.0)
Pneumonia	0	3 (1.0)
Acute respiratory failure	7 (2.3)	1 (0.3)
Pulmonary embolism	5 (1.7)	0
**Any serious AE related to treatment**	3 (1.0)	3 (1.0)
**Any fatal AE**	16 (5.3)	16 (5.3)
COVID-19	3 (1.0)	5 (1.7)
COVID-19 pneumonia	2 (0.7)	4 (1.3)
**Any fatal AE related to treatment**	0	0

Data are presented as n (%). SoC: standard of care. ^#^: ≥10% patients in either treatment group for AEs; ≥5% for AEs considered related to treatment; ≥1% for AEs leading to discontinuation, or serious, severe or fatal AEs.

## Discussion

The primary objective of the study was not met with respect to hospital discharge or recovery to no limitation of activity. The study was not powered to evaluate the three key secondary end-points related to disease progression but, although not reaching statistical significance, trends favouring the addition of SNG001 to SoC were observed for each of these measures, including a 26% relative risk reduction in patients progressing to severe disease or death in the SNG001 plus SoC group compared with placebo plus SoC. Furthermore, consistent with previous clinical studies, including those in patients with asthma or COPD [19–21], SNG001 was well tolerated and had a favourable safety profile.

Efficacy analyses were also performed in the per-protocol population, which excluded patients whose treatment deviated from the protocol in a way that may have impacted evaluations. The most common reason for exclusion was not receiving at least two full doses of study medication in the first 3 days. The relative risk reduction in the patients who progressed to severe disease or death was 36% in the per-protocol population rather than 26% in the ITT population, although this was not statistically significant. Furthermore, in the *post hoc* subgroup analyses, conducted in patients with baseline clinical parameters associated with increased severe COVID-19 risk, differences in favour of SNG001 plus SoC were more marked than in the per-protocol or ITT populations, with relative risk reductions in progression to severe disease or death ranging from 44.8% (not significant) to 69.9% (p=0.046) when patients were grouped by age, presence of comorbidities and poor respiratory function (oxygen saturation ≤92% and/or respiratory rate ≥21 breaths·min^−1^ while on supplemental oxygen). This potential clinically important effect therefore needs to be confirmed in a future study adequately powered to assess this end-point.

The lack of impact on recovery contrasts with the results from the phase II study of inhaled IFN-β in patients with COVID-19 [23], conducted at the beginning of the COVID-19 pandemic (March to May 2020) before any treatments that had been evaluated in randomised controlled studies were implemented as SoC. Thus, SPRINTER differed from the previous study in that 18% of the included patients had been fully vaccinated, and a large proportion were receiving corticosteroids (87%) and/or antivirals (19%). These improvements in SoC, together with changes in hospital practice, may have masked our ability to show a treatment effect on the primary end-point. One of the consequences of these changes is that patients were discharged from hospital more quickly in the current trial. While in the phase II study the median time to hospital discharge in the subgroup of patients who were receiving oxygen by mask or nasal prongs (*i.e.* matching the population recruited into SPRINTER) was 9 days in the placebo plus SoC group (data on file), it was 8 days in SPRINTER overall, decreasing further to 6 days in the UK sites (where the phase II study was conducted; data on file and supplementary figure S6). Improvements in SoC have also been reported by the RECOVERY Collaborative Group. Initially in the RECOVERY platform study, conducted in 2020, 28-day mortality was 23% in patients who received dexamethasone plus SoC [27], whereas in a later study, conducted in 2021, 28-day mortality in the SoC group (with 95% of patients receiving a corticosteroid such as dexamethasone) was 14% [28]. Similarly, the proportion of patients discharged from hospital within 28 days in these groups increased from 67% to 78% [27, 28].

The favourable safety and tolerability profile of SNG001 observed in the SPRINTER study was consistent with the previous studies in patients with COVID-19, asthma and COPD [19–21, 23]. A similar proportion of patients in the two treatment groups experienced adverse events, most of which were mild or moderate in severity and not considered either treatment-related or serious. In terms of serious adverse events, pulmonary embolism only occurred in the placebo plus SoC group, an observation that is interesting as incidence of coagulation events is well documented for patients hospitalised with COVID-19.

Given the observation, which would need to be confirmed in further studies, that patients with poor respiratory function may gain greater benefit from SNG001, a potential limitation of the study is that patients requiring noninvasive ventilation, high-flow nasal oxygen therapy, endotracheal intubation or invasive mechanical ventilation could not be dosed. However, the nebuliser can be used in different configurations that should enable these patients to be dosed in future studies (supported by appropriate dose selection studies, taking into account drug delivery to the lungs). In addition, the timing of initiation of IFN treatment has been the subject of debate, with suggestions that later initiation could be less effective. Patients were excluded from the study only if the prior duration of symptoms was ≥3 weeks (although a recent positive SARS-CoV-2 test was required and most patients had a duration of symptoms <10 days). In the previous phase II study, in which SNG001 was more effective than placebo, the median duration of symptoms at recruitment was similar to the current study [23]. This suggests there is a wide window for initiation of treatment with SNG001.

In conclusion, although the primary objective of the study was not met, there were signals in the key secondary end-points which suggest that SNG001, on top of SoC, may have prevented progression to severe disease (although differences were not statistically significant). In addition, SNG001 was well tolerated with a favourable safety profile, validating the route of administration. When combined with the results of the previous phase II study, these findings provide a rationale to continue investigating SNG001, not only in hospitalised patients with COVID-19 (in the context of ongoing virus evolution and likely emergence of new variants), but also more widely in patients with severe seasonal viral lung infections, due to the broad spectrum and variant agnostic antiviral activity of IFN-β.

## Supplementary material

10.1183/23120541.00605-2022.Supp1 **Please note:** supplementary material is not edited by the Editorial Office, and is uploaded as it has been supplied by the author.
Supplementary material 00605-2022.supplement
