# Supplementary material for: Nebulised interferon-β1a (SNG001) in hospitalised COVID-19: SPRINTER phase III study
Source: ERJ Open Res. 2023 Mar 27;9(2):00605-2022. doi: 10.1183/23120541.00605-2022 (PMC9790107; doi:10.1183/23120541.00605-2022)
Supplement: Supplementary file 1 [file 00605-2022.supplement.pdf]

Nebulised interferon beta1a (SNG001) in hospitalised COVID-19: SPRINTER Phase III Study

Phillip D Monk, Jody L Brookes, Victoria J Tear, Toby N Batten, Marcin Mankowski, Tatjana Adzic-Vukicevic, Michael G Crooks, Davinder PS Dosanjh, Monica Kraft, Christopher E Brightling, Felicity J Gabbay, Stephen T Holgate, Ratko Djukanovic, Tom MA Wilkinson, on behalf of the SPRINTER Study Group

Supplement

The SPRINTER Study Group

In addition to the authors and the investigators, the SPRINTER Study Group included the below people.

Affiliation	Title, name and highest degree
Synairgen Research Ltd., Southampton General Hospital, Southampton, UK	Mrs R Beegan BSc<br>Dr S Dudley PhD<br>Dr S Reynolds PhD
SEH Clinical Research Ltd, Sarisbury Green, Southampton, UK	Mrs S Hemmings BSc (Hons)
tranScrip Limited, Wokingham, UK	Dr Dominic Bowers MRCA<br>Dr Stephen P Jones PhD
MG Clinical Ltd, Crewe, UK	Ms Mandy Garrett MRPharmS

List of investigators

Title	First Name	Last Name	Address	Country
Dr	Ricardo	Leon de la Fuente	Hospital Papa Francisco - Hospital	Argentina
Dr	Gonzalo	Perez Marc	Hospital Militar Central Cirujano Mayor Cosme Argerich	Argentina
Dr	Marcelo Oscar	Silva	Hospital Universitario Austral	Argentina
Prof	Michel	Moutschen	CHU de Liège - Domaine Universitaire du Sart Tilman	Belgium
	Mathias	Leys	AZ Groeninge - Campus Kennedylaan	Belgium
Dr	Laura	Gaspard	CHR de la Citadelle - Site Citadelle - Infectiologie	Belgium
Dr	Philippe	Clevenbergh	CHU Brugmann	Belgium
Prof	Sabine	Allard	UZ Brussel - Infectiologie	Belgium
Mr	Felipe	Pizzol	Hospital Sao Jose	Brazil
Dr	José Francisco	Saraiva	Instituto de Pesquisa Clínica de Campinas	Brazil
Prof	Suzana	Lobo	Hospital de Base	Brazil
Mrs	Tiago	Simon	Instituto Mederi de Pesquisa e Saúde	Brazil
Dr	Cristiano	Devenci Vendrame	Clínica SUPERA	Brazil
Dr	Regis	Rosa	Hospital Moinhos de Vento	Brazil
Dr	Jaime	Gomez Ayala	FOSCAL	Colombia
Dr	Andres	Cadena Bonfanti	Clínica de la Costa Ltda.	Colombia
Dr	Fernando	Montoya Agudelo	Clinica de la Mujer	Colombia
Prof	François	Raffi	CHU De Nantes - Infectious Diseases	France
Prof	Jean-Benoit	Arlet	Hôpital Européen Georges-Pompidou - HEGP - Médecine Interne	France
Dr	Karine	Lacombe	CHU - HÃ´pital Saint-Antoine - Maladies Infectieuses	France
Prof	Olivier	EPAULARD	CHU de Grenoble - Hôpital Albert Michallon	France
Dr	Jade	Ghosn	Hopital Bichat - Infectious Diseases	France
Prof	Vincent	Dubée	CHU d'Angers	France
Prof	Stephan	Budweiser	RoMed Medical Center Rosenheim	Germany
Prof	Matthias	Ebert	Universit tsklinikum Mannheim	Germany
Dr	Simon	Herkenrath	Krankenhaus Bethanien gGmbH	Germany
Dr	Namdev	Jagtap	VishwaRaj Hospital	India
Dr	Anita	Mathew	Fortis Hospital Mulund - Internal Medicine	India
Dr	Yandrapati	Raju	King George Hospital	India
Dr	Anil	Kumar T	M S Ramaiah Medical College and Hospitals	India
Dr	Rajendran	Kannan	Saveetha Medical College & Hospital	India
Dr	Nirav	Bhalani	Rhythm Heart Institute	India
Dr	Prabhu	Subramani	Bangalore Medical College and Research Institute	India
Dr	Sunil	kumar	Acharya Vinoba Bhave Rural Hospital	India

Title	First Name	Last Name	Address	Country
Dr	Jayaraju	B. S.	JSS Medical College and Hospital	India
Dr	Milind	Vyawahre	Government Medical College Nagpur	India
Dr	Kailash	Rathi	Suyog Hospital	India
Dr	Dipak	Viradia	Unity Hospital	India
Dr	Ashish	Deshmukh	Oriion Citicare Super Speciality Hospital - Internal Medicine	India
Dr	Ami	Mayo	Assuta Ashdod University Hospital	Israel
Dr	Galia	Rahav	The Chaim Sheba Medical Center	Israel
Dr	Ronen	Ben Ami	Sourasky Tel Aviv Medical Center - Infectious Diseases	Israel
Dr	Dror	Marchaim	Assaf Harofeh Medical Center	Israel
Dr	Kamal	Abu Jabal	Ziv Medical Center	Israel
Dr	Khetam	Hussein	Rambam Health Care Campus	Israel
Prof	Raffaele	Bruno	IRCCS Policlinico San Matteo	Italy
Dr	Stefania	Piconi	PO A.Manzoni di Lecco, ASST Lecco	Italy
Prof	Ivan	Gentile	AOU Federico II - Malattie Infettive	Italy
Prof	Giuliano	Rizzardini	Ospedale Luigi Sacco, AO-PU	Italy
Dr	Guido	Chichino	PO Civile SS.Antonio e Biagio	Italy
Dr	Giovanna	Travi	Azienda Ospedaliera Niguarda Ca'Granda	Italy
Prof	Marco	Falcone	AOU Pisana	Italy
Dr	Paolo	Bonfanti	Azienda Socio Sanitaria Territoriale - ASST di Monza - Malattie Infettive	Italy
Dr	francesco	De Rosa	A.O.U. Città della Salute e della Scienza di Torino	Italy
Dr	Juan	Moreno Hoyos Abril	Fundación Santos y de la Garza Evia, I.B.P	Mexico
Dr	Jesus	Simon-Campos	Hospital General Dr. Agustin OˈHoran	Mexico
Dr	Jorge Alberto	Zamudio Lerma	Hospital General de Culiacan Dr. Bernardo J. Gastelum	Mexico
Dr	Juan Francisco	Rubio Suarez	EME RED Hospitalaria - COVID-19 ward	Mexico
Dr	Angel	Aleman Marquez	Clínica Sociedad Española de Beneficencia	Mexico
Dr	Martijn	Goosens	Gelre Ziekenhuis Zutphen	Netherlands
Dr	Jan Willem	van den Berg	Isala Klinieken	Netherlands
Dr	Stephanus	Gans	Ziekenhuis St Jansdal	Netherlands
Dr	Alexandre	Carvalho	Hospital de Braga	Portugal
Dr	Luis	Malheiro	C.H. de Vila Nova de Gaia/Espinho	Portugal
Dr	Helena	Sarmento	Hospital Senhora da Oliveira	Portugal
Dr	Gonçalo	Sarmento Costa	C Hosp de Entre Douro e Vouga	Portugal
Dr	Ines	Pintassilgo	Hospital Garcia de Orta	Portugal
Prof	Dragos	BUMBACEA	Spit Univ de Urgenta Elias	Romania
	Simin	FLORESCU	Sp Cl de Boli Inf si Trop V Babes	Romania
Prof	Cristian	Oancea	Sp Cl de Boli Infec si Pneumo V Babes	Romania
	Gheorghe	Diaconescu	Sp Cl de Boli Infec si Pneumo V Babes	Romania

Title	First Name	Last Name	Address	Country
Prof	Zorica	Lazic	Clinical Center Kragujevac	Serbia
Dr	Dusanka	Obradovic	Institute for Pulmonary Disease	Serbia
	Vesna	Turkulov	Clinical Center of Vojvodina	Serbia
Prof	Ivana	Stankovic	Clinical Center Nis	Serbia
Dr	Tatjana	Adzic Vukicevic	University Clinical Center of Serbia	Serbia
Dr	Jose Angel	Martín Oterino	Hospital Universitario de Salamanca	Spain
Dr	Enrique	Miguez Rey	CHU A Coruña	Spain
Dr	Vicente	Estrada Pérez	Hospital Clínico San Carlos	Spain
Dr	Santiago	Moreno Guillén	Hospital Universitario Ramón y Cajal	Spain
Mr	Francisco	Fanjul Losa	Hospital Universitario Son Espases	Spain
Dr	Alberto	Romero Palacios	Hospital Universitario de Puerto Real - Infectious disesases	Spain
Dr	Esther	Calbo Sebastián	Hospital Universitario Mutua de Terrassa	Spain
Dr	Pablo	Ryan	Hospital Universitario Infanta Leonor	Spain
Dr	Nicolas	Merchante	H U Nuesta Señora de Valme - Infectious Diseases	Spain
Dr	David	Ramos Barbón	Hospital Santa Creu i Sant Pau	Spain
Prof	Tom	Wilkinson	Southampton General Hospital	United Kingdom
Dr	Davinder	Dosanjh	Queen Elizabeth Hospital Birmingham	United Kingdom
Dr	Michael	Crooks	Castle Hill Hospital	United Kingdom
Dr	Christopher J	Duncan	Newcastle University - Institute of Cellular Medicine (ICM)	United Kingdom
Dr	David	Jackson	Guy's Hospital	United Kingdom
Dr	Najib	Rahman	Churchill Hospital	United Kingdom
Dr	Brendan	Healy	Morriston Hospital Swansea NHS Trust	United Kingdom
Dr	Robert	Willington	The Royal Bournemouth & Christchurch Hospitals NHS Foundation Trust	United Kingdom
Prof	Chris	Brightling	Glenfield General Hospital - Pharmacy Stores	United Kingdom
	Gemma	Hayes	Plymouth Hospitals NHS Trust - Derriford Hospital	United Kingdom
Dr	David	Chadwick	The James Cook University Hospital	United Kingdom
Dr	Hannah	Bayes	Glasgow Royal Infirmary	United Kingdom
Dr	Kanwal	Khalil	University Hospital of North Durham	United Kingdom
Dr	Omar	Touma	Wexham Park Hospital	United Kingdom
Dr	George	Evetts	Frimley Park Hospital	United Kingdom
Dr	Tom	Simpson	University Hospital Lewisham	United Kingdom

Title	First Name	Last Name	Address	Country
Dr	Dominick	Shaw	Nottingham University Hospitals NHS Trust	United Kingdom
Dr	Alison	Uriel	North Manchester General Hospital	United Kingdom
Dr	Raymond	Sheridan	Royal Devon & Exeter Hospital	United Kingdom
Dr	David	Brabham	PharmaTex Research, LLC	United States
Dr	John	McKinnon	Henry Ford Health System	United States
Dr	Linda	Rogers	Icahn School of Medicine at Mount Sinai (ISMMS) - The Mount Sinai Hospital (MSH)	United States
Dr	Monica	Kraft	Banner University of Arizona Medical Center	United States
Dr	Sameh	Hozayen	M Health Fairview University of Minnesota Medical Center	United States
Dr	Fadi	Saba	Professional Health Care of Pinellas	United States

Methods

Summary of protocol amendments

Table S1. Summary of protocol amendments.

Amendment number	Amendments	Date
1	Following discussions with regulatory authorities the study design was simplified, with one dose level of SNG001 tested instead of two. Under the previous design, SNG001 was to be administered at the current dose (the contents of two syringes), and at half the current dose (the contents of one syringe), with each SNG001 dose requiring administration of matching placebo. In addition to simplifying dosing, the removal of one dose level also decreased the number of patients required to be recruited. In addition, the order of the key secondary endpoints was altered, an interim analysis was introduced to test for futility, and an antigen test was included as evidence of positive SARS-CoV-2 status. Text was edited for clarity and consistency.	27 Nov 2020
2	Again, following discussions with regulatory agencies, time to hospital discharge was elevated from key secondary to primary endpoint (the study was already sufficiently powered, based on the assumptions for the existing primary endpoint, time to recovery), and the progression to intubation or death, and death secondary endpoints were elevated to key secondary endpoints (with a Hochberg procedure and gatekeeping strategy added to ensure the global alpha level was maintained).<br>The original sample size calculation was as follows: A sample size of approximately 610 patients in total using a 1:1 randomisation ratio would provide at least 90% power to detect a hazard ratio of 1.7 in time to recovery. This sample size was calculated using a global two-sided alpha level of 0.05 and allowed for an interim analysis to assess futility. This sample size assumed a recovery rate in the placebo treatment arm of 29% at Day 28 and a dropout rate of 25% spread uniformly over the 28-day study period.<br>The daily assessment of COVID-19 symptoms and limitation of usual activities was added as a secondary endpoint. In addition, the World Health Organization (WHO) Ordinal Scale of Clinical Improvement (OSCI) assessments were to continue until Day 35 (instead of Day 28). Text was edited for clarity and consistency.	21 Dec 2020
3	An exclusion criterion that prevented patients who had a previous SARS-CoV-2 vaccination from taking part in the study was removed. Text was edited for clarity and consistency.	22 Feb 2021
4	Additional guidance was provided on conducting various assessments, the importance of maintaining contact with patients throughout the 90-day follow-up period was emphasised, and additional guidance on the role of the Independent Data Monitoring Committee/Data Safety Monitoring Committee was provided. Text was edited for clarity and consistency.	9 Sep 2021

Inclusion criteria

1. Male or female, ≥18 years of age at the time of consent.
2. Admitted to hospital due to the severity of their COVID-19.
3. Positive virus test for SARS-CoV-2 using a validated molecular assay or validated antigen assay.

   Patients who had a positive virus test for SARS-CoV-2 prior to hospitalisation were to be randomised no later than 48 hours after hospital admission. If the virus test was performed more than 96 hours prior to hospitalisation, the test was to be repeated in the hospital prior to randomisation. Only patients whose repeated virus test is positive were randomised, no later than 48 hours after confirmation of SARS-CoV-2 infection.

   Patients who had their first positive virus test for SARS-CoV-2 after hospitalisation were randomised, no later than 48 hours after confirmation of SARS-CoV-2 infection.

4. Required oxygen therapy via nasal prongs or mask (WHO OSCI score of 4).
5. Provided informed consent.
6. Female patients were ≥1 year post-menopausal, surgically sterile, or using a defined highly effective method of contraception.
7. Women not of childbearing potential were defined as women either permanently sterilised (hysterectomy, bilateral oophorectomy, or bilateral salpingectomy), or who were postmenopausal.

   If, in the setting of the pandemic, the use of an acceptable birth control method was not possible, the decision to enrol a woman of childbearing potential was based on the benefit-risk for the patient, which was discussed with the patient at the time of the informed consent.

Exclusion Criteria

1. Evidence of ongoing SARS-CoV-2 infection for more than three weeks, confirmed by a validated molecular assay or validated antigen assay.

2. Non-invasive ventilation (continuous positive airway pressure/bilevel positive airway pressure) or high-flow nasal oxygen therapy (WHO OSCI score of 5).
3. Endotracheal intubation and invasive mechanical ventilation (WHO OSCI score of ≥6) or admission to intensive care.
4. Previous SARS-CoV-2 infection confirmed by a validated molecular assay or validated antigen assay.
5. Any condition, including findings in the patient's medical history or in the pre-randomisation study assessments that in the opinion of the investigator, constituted a risk or a contraindication for the participation of the patient into the study or that could interfere with the study objectives, conduct or evaluation.
6. Participation in previous clinical trials of SNG001.
7. Current or previous participation in another clinical trial where the patient received a dose of an investigational medicinal product (IMP) containing small molecules within 30 days or five half-lives (whichever is longer) prior to entry into this study or containing biologicals within 3 months prior to entry into this study.
8. Inability to use a nebuliser with a mouthpiece.
9. Inability to comply with the requirements for storage conditions of study medication in the home setting.
10. History of hypersensitivity to natural or recombinant interferon-β or to any of the excipients in the drug preparation.
11. Females who were breast-feeding, lactating, pregnant or intending to become pregnant.

WHO OCSI

The WHO OSCI is a nine-point scale (0, no clinical or virological evidence of infection; 8, death) as described in the February 2020 WHO R&D Blueprint for Novel Coronavirus [1], and was assessed either face-to-face or by telephone/video link by a clinically qualified member of the study team.

<table>
<tr><th>Patient State</th><th>Descriptor</th><th>Score</th></tr>
<tr><td>Uninfected</td><td>No clinical or virological evidence of infection</td><td>0</td></tr>
<tr><td rowspan="2">Ambulatory</td><td>No limitation of activities</td><td>1</td></tr>
<tr><td>Limitation of activities</td><td>2</td></tr>
<tr><td rowspan="2">Hospitalised</td><td>Hospitalised, no oxygen therapy</td><td>3</td></tr>
<tr><td>Oxygen by mask or nasal prongs</td><td>4</td></tr>
<tr><td rowspan="3">Hospitalised</td><td>Non-invasive ventilation or high-flow oxygen</td><td>5</td></tr>
<tr><td>Intubation and mechanical ventilation</td><td>6</td></tr>
<tr><td>Ventilation + additional organ support – pressors, renal replacement therapy (RRT), extracorporeal membrane oxygenation (ECMO)</td><td>7</td></tr>
<tr><td>Dead</td><td>Death</td><td>8</td></tr>
</table>

To allow a consistent approach to the OSCI assessment for patients that were discharged from hospital, on the day of hospital discharge and on the days following hospital discharge patients were asked the following questions about their clinical status and return to the pre-COVID-19 level of activity:

- “In the past 24 hours, did you experience any signs or symptoms of your coronavirus infection?” (Yes/No)
- “In the past 24 hours, did you feel that your usual activities (e.g. work, study, housework, family or leisure activities) have returned to the level from before your coronavirus infection and did not require additional assistance/support*?” (Yes/No)

*Assistance/support was defined as additional help of other people and/or requirement for supplemental oxygen (or a higher level of supplemental oxygen), compared to the pre-COVID-19 state.

To minimise any potential influence on the patients, trial staff read the questions to patients verbatim. The below scoring algorithm was applied.

**Presence of signs/symptoms of coronavirus infection (or virological evidence of infection)?**	**Usual activities returned to baseline level?**	**WHO OSCI score**
No	Yes	0
Yes	Yes	1
No	No	2
Yes	No	2

BCSS

Patients were asked by trained staff to report the severity of breathlessness, cough and sputum symptoms, each on a five-point scale with higher scores indicating more severe symptoms [2].

1. How much difficulty did you have breathing today?
   - 0 = None – unaware of any difficulty
   - 1 = Mild – noticeable when performing strenuous activity (e.g. running)
   - 2 = Moderate – noticeable even when performing light activity (e.g. bedmaking or carrying groceries)
   - 3 = Marked – noticeable when washing or dressing
   - 4 = Severe – almost constant, present even when resting
2. How was your cough today?
   - 0 = No cough – unaware of coughing
   - 1 = Rare – cough now and then
   - 2 = Occasional – less than hourly
   - 3 = Frequent – one or more times an hour
   - 4 = Almost constant – never free of cough or need to cough
3. How much trouble did you have due to sputum today?
   - 0 = None – unaware of any trouble
   - 1 = Mild – rarely caused trouble
   - 2 = Moderate – noticeable trouble
   - 3 = Marked – caused a great deal of trouble
   - 4 = Severe – almost constant trouble

NEWS2

NEWS2 is a tool developed by the Royal College of Physicians that aggregates physiological measurements which are already recorded in routine practice [3]. The highest NEWS2 score for each calendar day was collected; data were not recorded after discharge.

Six simple physiological parameters form the basis of the scoring system:

1. Respiration rate
2. Oxygen saturation
3. Any supplementary oxygen
4. Temperature
5. Systolic blood pressure
6. Heart rate
7. Alert, Voice, Pain, Unresponsive.

COVID-19 symptom assessment

Fever/feeling feverish	◯ Yes	◯ No	Wheezing	◯ Yes	◯ No
Cough	◯ Yes	◯ No	Chest Pain	◯ Yes	◯ No
Cough with sputum	◯ Yes	◯ No	Muscle aches (myalgia)	◯ Yes	◯ No
Cough with bloody sputum/haemoptysis	◯ Yes	◯ No	Joint pain (athralgia)	◯ Yes	◯ No
Sore throat	◯ Yes	◯ No	Fatigue/ malaise	◯ Yes	◯ No
Runny nose (rhinorrhoea)	◯ Yes	◯ No	Shortness of Breath (dysponea)	◯ Yes	◯ No
Ear pain	◯ Yes	◯ No	Loss of smell and/or taste	◯ Yes	◯ No
Headache	◯ Yes	◯ No	Vomiting/nausea	◯ Yes	◯ No
Other	◯ Yes	◯ No			

If 'other', specify here:

Statistical methods

Covariate-adjusted differences in proportions were derived from the logistic model estimates, and were formally assessed for statistical significance with a gatekeeping strategy. The proportions of patients recovering, or discharged from hospital were assessed using logistic regression models, and the improvement in OSCI score was assessed using ordinal logistic regression models. The change in BCSS was assessed using mixed models for repeated measures (MMRM). All other secondary endpoints were summarised descriptively only. For the handling of missing data see the supplement.

For the primary endpoints, patients who died were censored at 28 days, the maximum time to event allowed by the study design (note that as hospital discharge or recovery had to be sustained for at least 7 days, the latest timepoint at which a patient could be discharged or recover to be considered in the primary endpoints was Day 28). For analyses using the WHO OSCI, including the primary and key secondary endpoints, patients with a WHO OSCI score of 8, indicating death, had subsequent missing WHO OSCI assessments imputed as 8. In addition, if other data sources such as adverse events indicated a patient died, all missing WHO OSCI scores on and after the date of death were imputed as 8. Other missed OSCI assessments were not imputed for the primary endpoints, but hospital discharge was confirmed by the patient's location, and recovery was only confirmed if sufficient non-missing data were available. Patients who could not be confirmed as discharged/recovered or who withdrew from the study within 7 days of the event were treated as censored at the date last known to be hospitalised/not recovered. Key secondary endpoints were derived using observed data. For BCSS, missing breathless scores were imputed as 4 if the WHO OSCI score at the corresponding visit was ≥5, with cough and sputum scores considered missing at random and total scores calculated by summing the imputed symptom scores, where possible. Missing breathlessness, cough and sputum scores at all other visits were considered missing at random and were imputed, but were accounted for by the MMRM analysis.

Results

Table S2. Reasons for exclusion from the per-protocol population.

Reason	Treatment group	
	**Placebo**	**SNG001**
Clinical practice had a potential impact on efficacy assessment	1	1
Patient or relatives declined advanced respiratory support	0	3
Failed to receive two full doses of study medication in the first three days of treatment	36	31
Discharged from hospital for reason other than severity of condition	15	16
No positive SARS-CoV-2 result	0	1
Patient first reported symptoms more than three weeks prior to randomisation	1	0
Received study medication that was outside of temperature range	0	1

Table S3. Proportions of patients recovering or discharged from hospital, and changes from baseline in BCSS score (intention-to-treat population).

**Parameter**	**Placebo plus SoC (N=314)**	**SNG001 plus SoC (N=309)**	**SNG001 vs placebo difference**
Patients recovering (WHO OSCI score ≤1 sustained for ≥7 days)			
At Day 7	17 (5.4%)	28 (9.1%)	1.71 (0.90, 3.22); 0.101
At Day 14	73 (23.2%)	75 (24.3%)	0.99 (0.67, 1.45); 0.942
At Day 21	118 (37.6%)	117 (37.9%)	0.96 (0.68, 1.35); 0.824
At Day 28	151 (48.1%)	145 (46.9%)	0.92 (0.66, 1.28); 0.613
Patients discharged from hospital			
At Day 7	141 (44.9%)	154 (49.8%)	1.18 (0.85, 1.64); 0.323
At Day 14	223 (71.0%)	231 (74.8%)	1.17 (0.81, 1.70); 0.406
At Day 21	249 (79.3%)	245 (79.3%)	0.96 (0.64, 1.43); 0.828
At Day 28	255 (81.2%)	249 (80.6%)	0.92 (0.61, 1.40); 0.706
Change from baseline in BCSS total score			
At Day 7	–2.2 (–2.4, –2.0)	–2.1 (–2.3, –1.9)	0.1 (–0.3, 0.4); 0.726
At Day 14	–3.0 (–3.2, –2.8)	–2.9 (–3.1, –2.6)	0.2 (–0.2, 0.5); 0.354
Days 2–15	–2.2 (–2.4, –2.1)	–2.1 (–2.3, –2.0)	0.1 (–0.1, 0.3); 0.410
Change from baseline in BCSS breathlessness score			
At Day 7	–0.71 (–0.83, –0.59)	–0.75 (–0.88, –0.63)	–0.04 (–0.22, 0.13); 0.627
At Day 14	–0.99 (–1.12, –0.86)	–1.03 (–1.16, –0.91)	–0.04 (–0.22, 0.14); 0.635
Days 2–15	–0.75 (–0.85, –0.65)	–0.78 (–0.87, –0.68)	–0.03 (–0.17, 0.11); 0.699
Change from baseline in BCSS cough score			
At Day 7	–0.93 (–1.03, –0.82)	–0.84 (–0.94, –0.73)	0.09 (–0.06, 0.24); 0.255
At Day 14	–1.31 (–1.42, –1.21)	–1.17 (–1.28, –1.07)	0.14 (–0.01, 0.29); 0.065
Days 2–15	–0.93 (–1.01, –0.86)	–0.84 (–0.91, –0.76)	0.10 (–0.01, 0.20); 0.063
Change from baseline in BCSS sputum score			
At Day 7	–0.44 (–0.52, –0.37)	–0.43 (–0.50, –0.35)	0.02 (–0.09, 0.12); 0.757

Parameter	Placebo plus SoC (N=314)	SNG001 plus SoC (N=309)	SNG001 vs placebo difference
At Day 14	–0.60 (–0.67, –0.53)	–0.56 (–0.63, –0.48)	0.05 (–0.06, 0.15); 0.377
Days 2–15	–0.46 (–0.51, –0.40)	–0.42 (–0.48, –0.37)	0.04 (–0.04, 0.11); 0.350

Treatment group data are number of patients (%) or least squares mean (95% confidence interval). SNG001 vs placebo differences are odds ratio (95% CI); p value, except for BCSS endpoints, which are least squares mean (95% confidence interval); p value. SoC, standard of care; WHO OSCI, World Health Organization Ordinal Scale of Clinical Improvement; BCSS, Breathlessness, Cough and Sputum Scale.

Figure S1. Patients categorised by WHO OSCI score at baseline and Days 7, 14, 21 and 28, with odds ratio for a better outcome (intention-to-treat population).

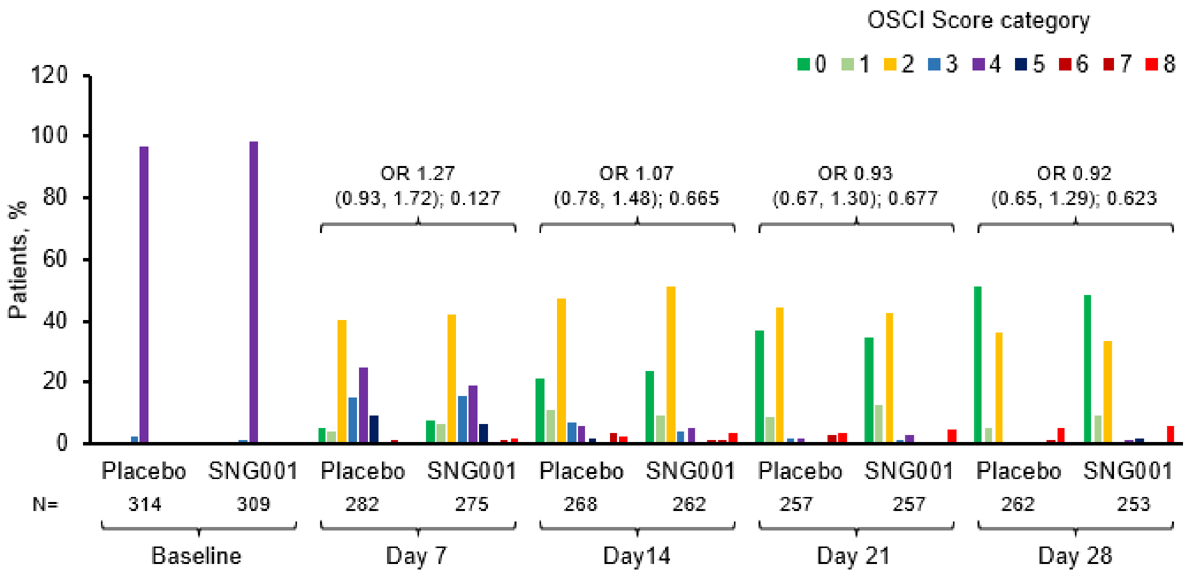

WHO OSCI, World Health Organization Ordinal Scale of Clinical Improvement (0 = No clinical or virological evidence of infection; 1 = No limitation of activities; 2 = Limitation of activities; 3 = Hospitalised – no oxygen therapy; 4 = Oxygen by mask or nasal prongs; 5 = Non-invasive ventilation, or high flow oxygen; 6 = Intubation and mechanical ventilation; 7 = Ventilation plus additional organ support; 8 = Death); OR, odds ratio.

Figure S2. Change from baseline in Breathlessness, Cough and Sputum Scale.

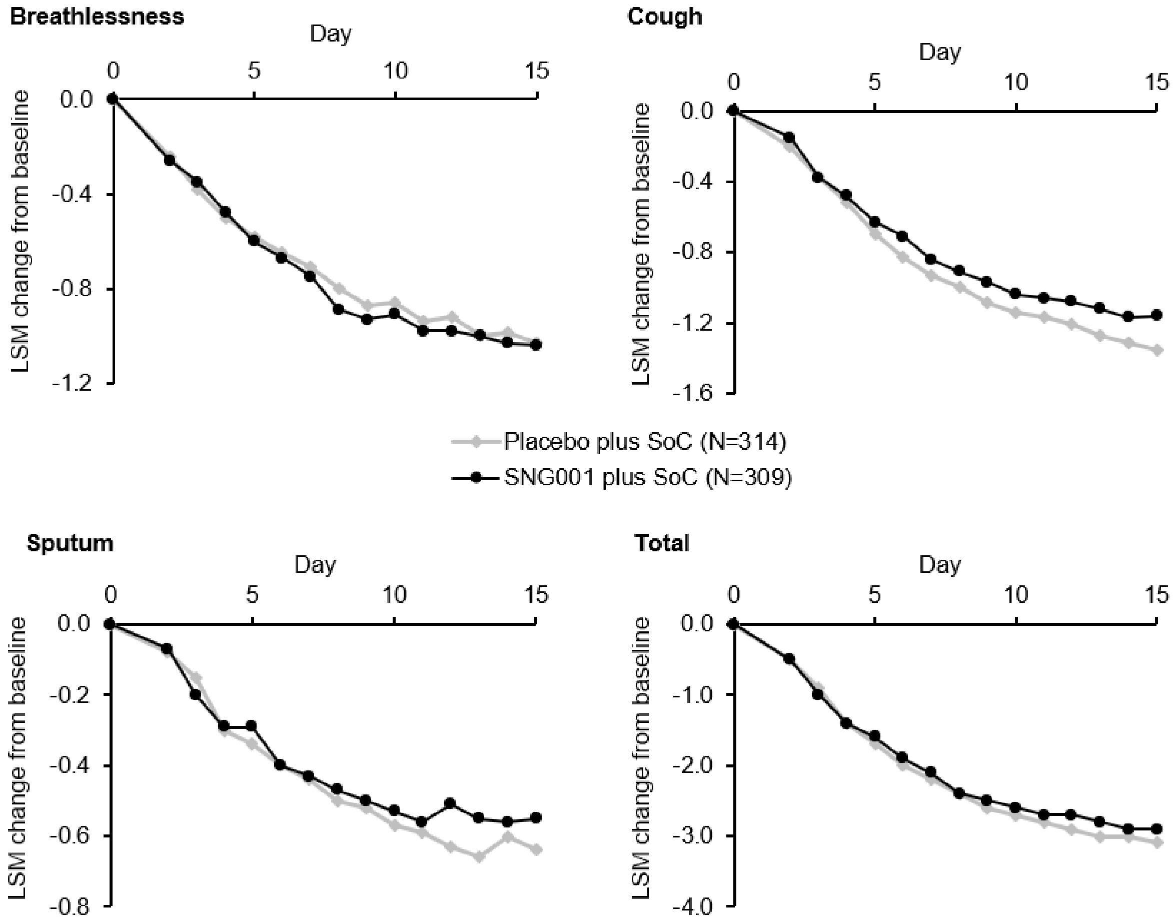

SoC, standard of care; LSM, least squares mean.

Figure S3. Mean change from baseline in National Early Warning System-2 score during the hospitalisation period.

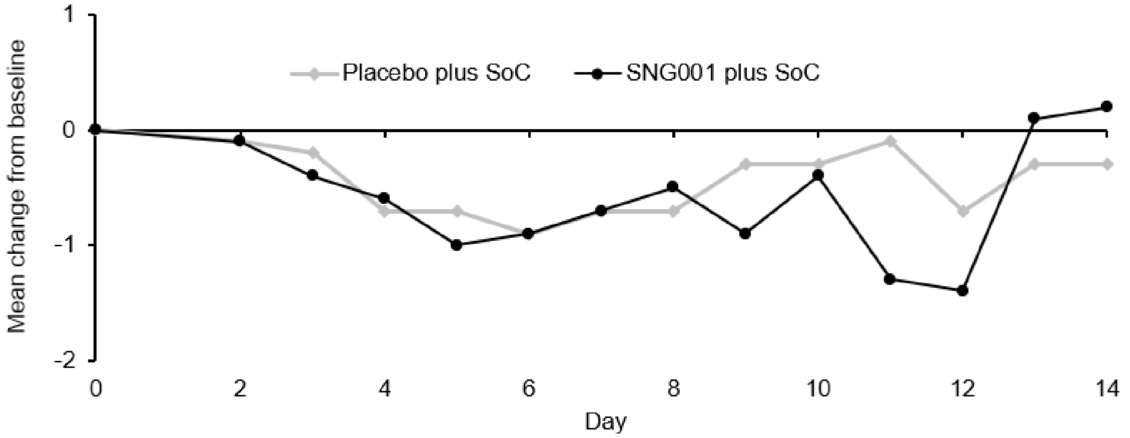

Placebo N=305	182	171	141	116	103	87	74	57	47	43	40	36	30
SNG001 N=302	181	168	150	127	97	75	55	46	38	35	28	21	17

Note: Data are presented up to Day 14 only. After this timepoint, too few patients have available data for meaningful interpretation of the results (mainly due to hospital discharge). SoC, standard of care.

Figure S4. Proportion of patients with any COVID-19 related symptom.

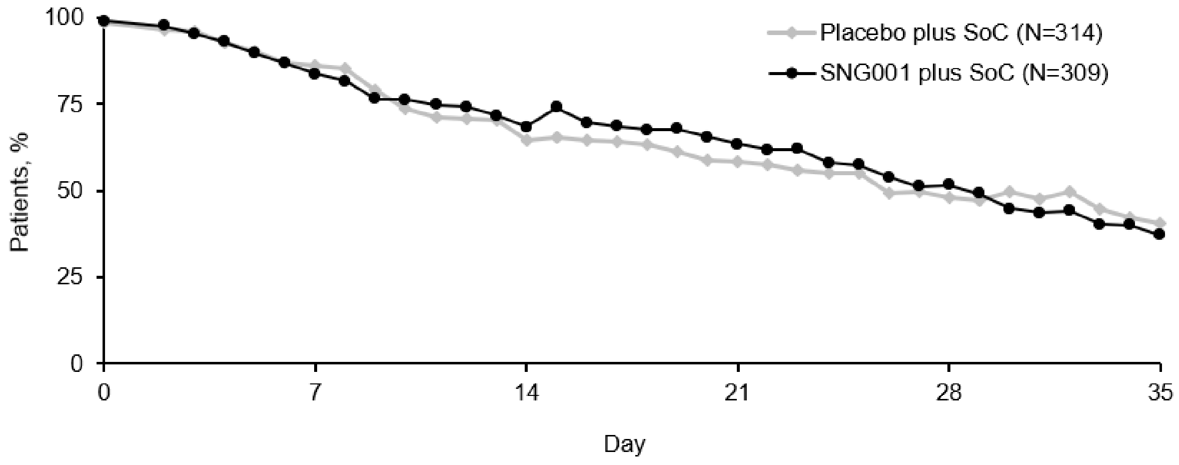

SoC, standard of care.

Table S4. EuroQol 5-dimension 5-level change from baseline (intention-to-treat population).

Parameter	Placebo plus SoC (N=314)	SNG001 plus SoC (N=309)
UK Crosswalk Index		
At Day 7	0.14 (0.293)	0.14 (0.267)
At Day 15 (end of treatment)	0.21 (0.288)	0.24 (0.253)
At Day 28 (follow-up)	0.28 (0.282)	0.26 (0.266)
Visual analogue scale		
At Day 7	13.1 (18.53)	15.8 (19.96)
At Day 15 (end of treatment)	20.9 (19.17)	22.8 (19.87)
At Day 28 (follow-up)	24.8 (20.84)	26.7 (20.26)

Data are mean (standard deviation). SoC, standard of care.

Figure S5. Mean change from baseline in EuroQol 5-dimension 5-level usual activities subscale.

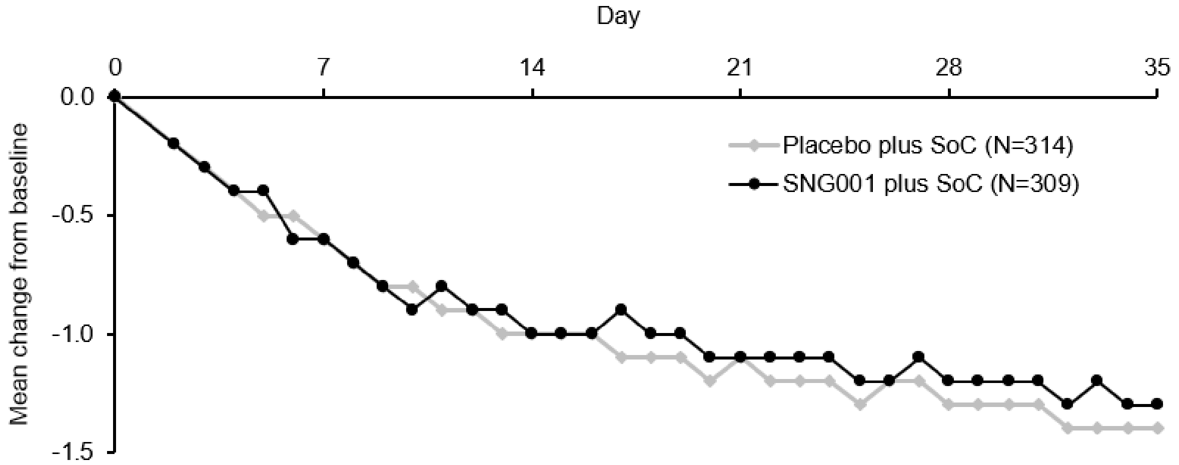

SoC, standard of care.

Figure S6. Time to hospital discharge in the placebo plus standard of care groups in UK sites of the current study, and in the previous Phase II study.

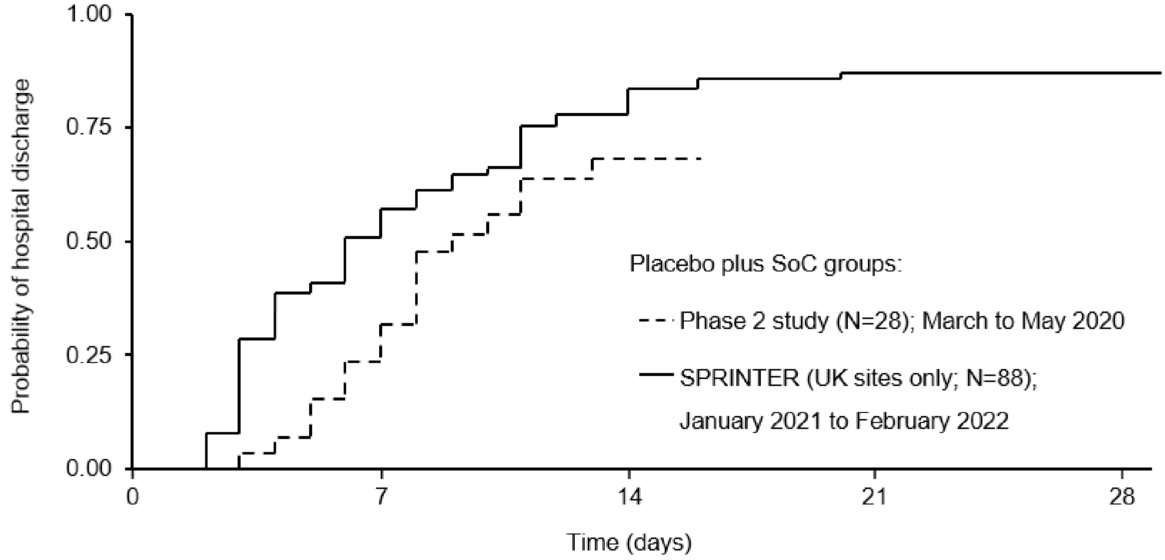

SoC, standard of care.

References

1. World Health Organization. WHO R&D Blueprint: Novel coronavirus [Internet]. 2020 [cited 2022 Jul 27].Available from: https://www.who.int/publications/i/item/covid-19-therapeutic-trial-synopsis.
2. Leidy NK, Rennard SI, Schmier J, Jones MKC, Goldman M. The Breathlessness, Cough, and Sputum Scale. The development of empirically based guidelines for interpretation. Chest 2003; 124: 2182–2191.
3. Royal College of Physicians. National Early Warning Score (NEWS) 2: Standardising the assessment of acute-illness severity in the NHS. Updated report of a working party. 2017.
